# Electrochemical Immunoassay Using Open Circuit Potential Detection Labeled by Platinum Nanoparticles

**DOI:** 10.3390/s18020444

**Published:** 2018-02-03

**Authors:** Kanokwan Charoenkitamorn, Phan Trong Tue, Keiko Kawai, Orawon Chailapakul, Yuzuru Takamura

**Affiliations:** 1 School of Materials Science, Japan Advanced Institute of Science and Technology, 1-1 Asahidai, Nomi City, Ishikawa 923-1292, Japan; k.charoenkitamorn@jaist.ac.jp (K.C.); phan-tt@jaist.ac.jp (P.T.T.); keiko-k@jaist.ac.jp (K.K.); 2 Department of Chemistry, Faculty of Science, Chulalongkorn University, 254 Phayathai Road, Patumwan, Bangkok 10330, Thailand; 3 National Center of Excellent of Petroleum, Petrochemicals and Advanced Materials, Chulalongkorn University, Patumwan, Bangkok 10330, Thailand

**Keywords:** human chorionic gonadotropin, screen-printed carbon electrode, platinum nanoparticles, open circuit potential, electrochemical immunosensor

## Abstract

In this work, a simple electrochemical immunoassay based on platinum nanoparticles (PtNPs) using open circuit potential (OCP) detection was developed. The detection of human chorionic gonadotropin hormone (hCG) as a model analyte, was demonstrated by direct electrical detection of PtNPs in hydrazine solution using OCP measurement without any application of either potential or current to the system. Disposable screen-printed carbon electrodes (SPCEs) were utilized for the development of our immunosensor, which required a sample volume as small as 2 μL. After preparation of a sandwich-type immunosystem, hydrazine solution was dropped on the electrode’s surface, which was followed immediately by electrical detection using OCP. The change of the OCP signal originated from electrocatalytic oxidation of the hydrazine on PtNPs. Under the optimal conditions of a pH of 6.0 and a hydrazine concentration of 1 mM, a detection limit of 0.28 ng mL^−1^ and a linearity of 0–10 ng mL^−1^ were obtained. The PtNP-based OCP method is a simpler electrochemical detection procedure than those obtained from other electrochemical methods and has an acceptable sensitivity and reproducibility. The simplicity of the detection procedure and the cost-effectiveness of the disposable SPCE illustrate the attractive benefits of this sensor. Moreover, it could be applied to a simplified and miniaturized diagnostic system with minimal user manipulation.

## 1. Introduction

Recently, considerable attention has been paid to point-of-care (POC) diagnostic techniques that can be performed in the clinic. There is an increasing requirement for the development of simple, reliable, and ultrasensitive biosensors with rapid response for the detection of protein biomarkers. Electrochemical sensors represent an important and continuously growing analytical field because of their simplicity of fabrication, low cost of instrumentation, scope for mass fabrication, short response time, and high sensitivity [1,2,3,4,5,6]. Electrochemical methods have received significant interest for the development of selective and sensitive biosensors that could detect substances of great interest at concentration <10^−6^ M in applications as varied as clinical analysis and diagnostics, food safety control, drug screening and development, environmental monitoring, forensic analysis, and the management of biological threats [4,7,8]. 

For measurement of bio-substances at low concentration ranges less than ng mL^−1^, immunoassay based on amperometry is advantageous for its sensitivity and selectivity. Many researchers have developed voltammetry- and amperometry-based immunosensors for single target in measurement range from ng mL^−1^ to pg mL^−1^ [9,10,11]. 

These types of sensors, however, still have some drawbacks. One of them is that they are not easily used for multiple detection, which is sometimes required for clinical application. For multiplex detection by the electrochemical method, multiple working electrodes system can be considered. In such a system, interference between working electrodes becomes a big problem. To solve this problem, one of the ways is just to repeat the voltammetry for each working electrodes one by one, but it consumes long measurement time. Another drawback is that the amperometric and voltammetric methods require voltage supply source to apply and to scan the potential in a complicated manner. In this regard, open circuit potential (OCP) appears to be an attractive detection principle for the development of simple biosensors [12,13,14]. 

OCP measures the different potentials between working electrodes immersed in medium solution and a suitable reference electrode with the application of neither potential nor current. Compared to voltammetry and amperometry, the OCP technique possesses several advantages, such as a two-electrode mode including working and reference electrodes, spontaneous measurement of the electrode potential built by electrochemical reactions on the electrode surface, potential for miniaturization of the reference electrode, and easy acquisition of multiple electrode potentials at once. Moreover, since OCP measures all redox reactions without the application of any voltage, there is less interference by contaminants [15]. Thus, OCP can be applied to simplify and miniaturize electrochemical systems for using either in laboratories or in the field for clinical diagnosis. 

Recently, several researchers have presented the development of OCP-based biosensors using enzymes as electrochemical labels for the determination of various kinds of protein biomarkers. Ahammad and co-workers [16] have developed gold-nanoparticle-modified indium tin oxide electrodes for immobilizing a horseradish peroxidase (HRP)-conjugated antibody and applied it to the detection of the cardiac biomarker, troponin I, by an OCP sensor. The OCP signal resulted from the catalytic reduction of HRP on the surface. This sensor provided the sensitivity in the range of 1–100 ng mL^−1^. The design of an OCP-based biosensor for glucose monitoring has been proposed by Song and co-authors [15]. A Kenaf stem-derived macroporous carbon microelectrode was used to load glucose oxidase. The electrocatalysis oxidation of glucose on the electrode surface directly resulted in a change of the OCP signal. The detection limit (LoD) was observed to be 10 μM (1.8 μg mL^−1^). Wong et al. [17] have reported the development of a label-free OCP-based system for the detection of prostate cancer biomarkers. They used an electronic amplifier system setup to provide an accurate measure of OCP variation. DNA aptamers were employed against prostate-specific antigens. This sensor could differentiate 0.1 ng mL^−1^ of prostate-specific antigen from a blank measurement. As the illustrative examples given indicate, the OCP method offers a highly sensitive yet simple manipulation procedure. However, most of OCP based biosensors have employed the use of enzymatic labeled, which are less stable and vulnerable to degradation/denaturation caused by the solution matrix. Therefore, the development of more versatile and stable immunoassays for protein biomarker detection is highly desired.

Recently, metal nanoparticles have been initiated as an alternative to the enzymes in biosensors. Compared to an enzyme, metal nanoparticles show better long-term stability. In this context, the oxidation of hydrazine solution by platinum nanoparticles (PtNPs) due to their excellent electrocatalytic activity [18] makes the combination a promising candidate for OCP-based biosensors. In this work, we are interested in the use of PtNPs in a hydrazine solution of redox molecules because the oxidation of hydrazine is well-electrocatalyzed at the PtNPs, thereby making the OCP sensor more sensitive. We first propose a new simple OCP-based electrochemical immunoassay using the PtNPs as an electrochemical label for the determination of human chorionic gonadotropin hormone (hCG), as a model analyte. The detection of hCG was demonstrated by direct electrical detection of the PtNPs in a hydrazine solution using OCP measurements without the application of any external potential. The change of the OCP signal originated from the electrocatalytic oxidation of the hydrazine on the PtNPs. Our proposed method shows simpler electrochemical detection procedures than those obtained from voltammetry and amperometry based detection with an acceptable sensitivity and reproducibility. Moreover, it could be applied in a simplified and miniaturized diagnostic system with minimal user manipulation.

## 2. Materials and Methods

### 2.1. Chemicals and Reagents

Monoclonal anti-human α-subunit of follicle-stimulating hormone (Mab-FSH) with an affinity constant of 2.8 × 10^−9^ M^−1^, and monoclonal anti-human chorionic gonadotropin (Mab-hCG) with an affinity constant of 4.9 × 10^−9^ M^−1^, were purchased from Medix Biochemica (Kauniainen, Finland). A colloidal solution of Au nanoparticles of diameter 40 nm was purchased from Tanaka Kikinzoku Kogyo K. K. (Tokyo, Japan). Bovine serum albumin (BSA) was purchased from Sigma-Aldrich, (St. Louis, MO, USA). Hydrazine (N_2_H_4_), disodium hydrogen phosphate (Na_2_HPO_4_), sodium dihydrogen phosphate (NaH_2_PO_4_), polyethylene glycol (PEG), and potassium dihydrogen phosphate (KH_2_PO_4_) were purchased from Wako Pure Chemical Industries (Osaka, Japan). Sodium azide (NaN) was purchased from Nacalai Tesque (Kyoto, Japan). All chemicals used in this work were of analytical grade, and all solutions were prepared using Milli-Q water from Millipore (R ≥ 18. 2 M Ω cm^−1^).

### 2.2. Instruments

The OCP measurements were performed on a potentiostat model PGSTAT 100 (Metrohm U.K. Ltd., Cheshire, UK). The planar screen-printed carbon electrodes (SPCEs) were kindly donated by BioDevice Technology Ltd. (Ishikawa, Japan). The SPCE consisted of three electrodes, including the carbon working electrode, carbon counter electrode, and silver/silver chloride reference electrode with a total length of 11 mm, and a geometric working area of 2.64 mm^2^. Scanning electron microscope (SEM) images of the SPCE surfaces were obtained using a VE-7800 (Keyence, Japan) at an acceleration voltage of 20 kV, and Hitachi S-4500 in-lens cold-cathode field-emission ultrahigh-resolution SEM.

### 2.3. Sandwiched Immunosensor Procedure

#### 2.3.1. Preparation of PtNP-Labeled hCG Antibody (Pt-Mab-hCG)

The preparation of Pt-Mab-hCGs was performed using slight modification to the method previously reported by our group [9,19]. Briefly, 200 μL of an aliquot of Mab-hCG solution (50 μg/mL in 5 mM KH_2_PO_4_, pH 7.5) was mixed with 1.8 mL of 0.1% PtNP solution, and kept for 10 min at room temperature. Then, the uncoated surfaces of the PtNPs were prevented using 100 μL of 1% PEG in 50 mM KH_2_PO_4_ solution (pH 7.5) and 200 μL of 10% BSA in 50 mM KH_2_PO_4_ solution (pH 9.0) as a blocking solution. After the immobilization and blocking procedures, PtNP-conjugated Mab-hCGs (Pt-Mab-hCGs) were collected by centrifugal operation (8000 g for 15 min at 4 °C). Pt-Mab-hCGs were suspended in 2 mL of the preservation solution consisting of 1% BSA, 0.05% PEG 20,000, 0.1% NaN_3_, and 150 mM NaCl in 20 mM Tris-HCl buffer, pH 8.2, and collected after centrifuging under the same conditions. For the stock solution, Pt-Mab-hCGs were suspended in 200 µL preservation solution.

#### 2.3.2. Immobilization of Primary Monoclonal Antibodies on the Working Electrode Surface of SPCE (Mab-FSH-Immobilized Immunosensor)

In order to immobilize Mab-FSH on the working electrode, the SPCEs were exposed overnight (at 4 °C) to 100 µg mL^−1^ Mab-FSH solution in 50 mM phosphate buffer (pH 7.4) by dropping 2.0 μL of Mab-FSH on the working electrode surface of SPCE. Following this, the excess antibodies were rinsed with PBS. To suppress nonspecific binding, 2.0 μL of 50 mM PBS solution containing 1% BSA was added to the resulting electrodes, and incubated for 12 h at a controlled temperature of 4 °C, to prevent denaturation of the BSA during the process. Finally, the electrodes were rinsed with PBS and maintained at 4 °C until further use.

#### 2.3.3. Immobilization of hCG Antigens and Pt-Mab-hCG on the Mab-FSH-Immobilized Immunosensor

**Figure 1:**
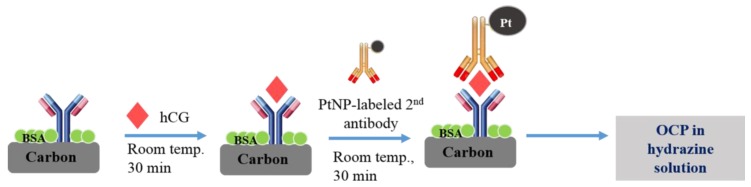
Schematic illustration of the immobilization of human chorionic gonadotropin hormone (hCG) antigens and platinum nanoparticle (PtNP)-Labeled hCG Antibody (Pt-Mab-hCG) onto Mab-FSH-immobilized immunosensor.

A scheme illustrating the principle of the PtNPs-based OCP immunoassay on the SPCE is shown in Figure 1. Different concentrations of the hCG antigen solution were made by diluting the stock solution in PBS containing 1% BSA for detection. These sample solutions were introduced to the Mab-FSH-immobilized working electrode of SPCEs for 30 min at room temperature. After rinsing with PBS, 2.0 μL of Pt-Mab-hCG solution was applied to the surface and incubated for 30 min at room temperature. Finally, the SPCEs were rinsed carefully with PBS.

### 2.4. Electrochemical System

**Figure 2:**
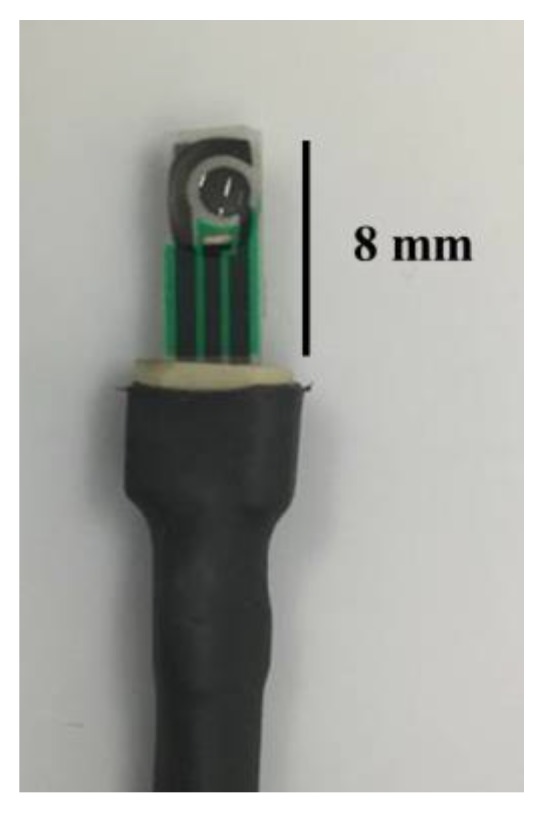
Schematic illustration of commercial screen-printed carbon electrode (SPCE).

The detection procedure was performed using a potentionstat system. First, the direct electrocatalytic reaction was performed using 1 mM hydrazine solution in 50 mM PBS pH 6.0, which covered the two-electrode zone of the SPCE at room temperature, immediately followed by OCP measurement for 200 s, as shown in Figure 2.

## 3. Results and Discussion

### 3.1. Surface Morphology of Sandwich-Type Immunosensors Labeled with the PtNPs

**Figure 3:**
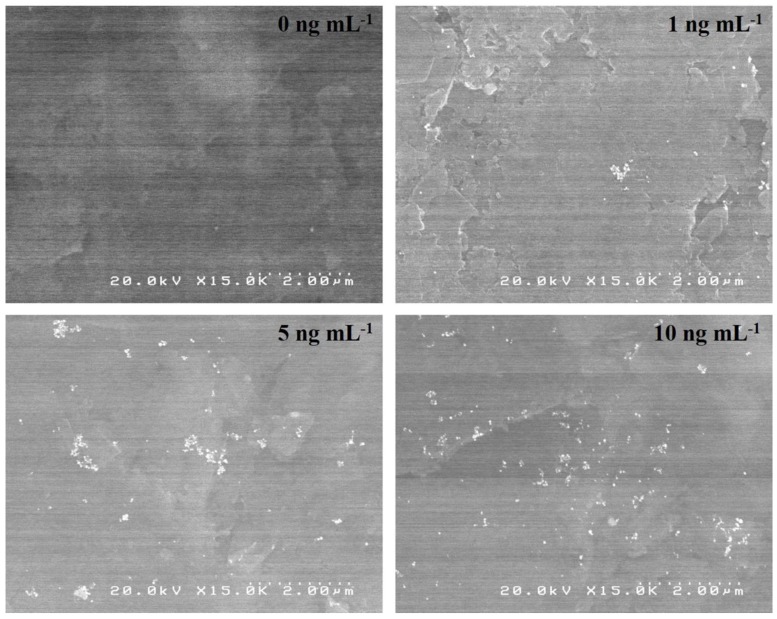
SEM images of the electrode surface of PtNP-labeled immunocomplexes immobilized on SPCE surface at the concentration of 0, 1, 5, and 10 ng mL^−1^ of hCG.

Surface morphologies of the PtNPs-labeled immunocomplexes, consisting of primary Mab, hCG, and PtNP-labeled secondary Mab, were evaluated by the SEM, as shown in Figure 3. Different hCG concentrations, ranging between 0 and 10 ng mL^−1^, were investigated. The PtNPs were clearly observed at the electrode surface which confirmed that PtNP-labeled secondary Mab was successfully immobilized on the immunosensor. The number of PtNP-labeled secondary antibody increased with increasing concentration of hCG. The size of the PtNPs was approximately 40 nm in diameter, which is consistent with the original platinum colloidal solution.

### 3.2. OCP Behavior of PtNPs Based Immunoassay for hCG Detection

**Figure 4:**
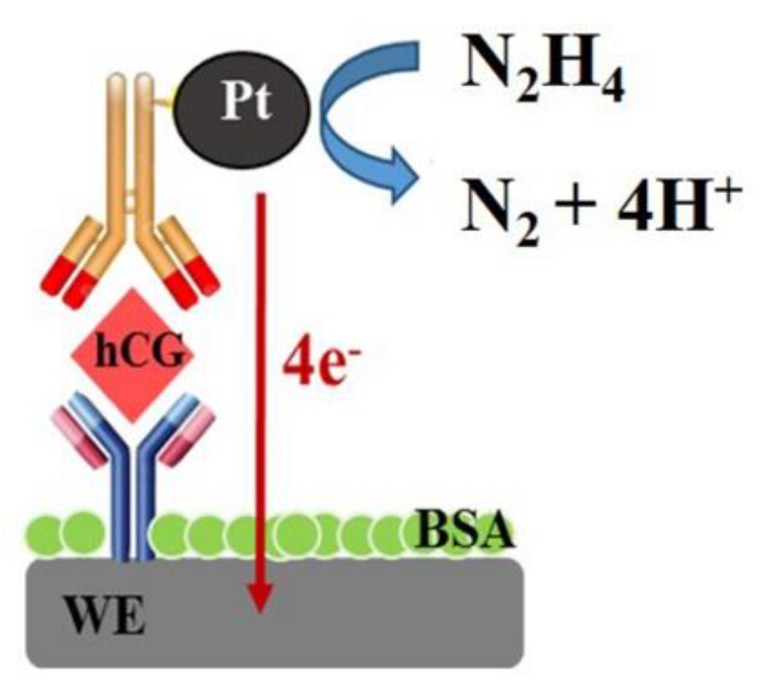
Schematic illustration of the electrocatalysis of hydrazine by PtNPs at secondary antibody.

**Figure 5:**
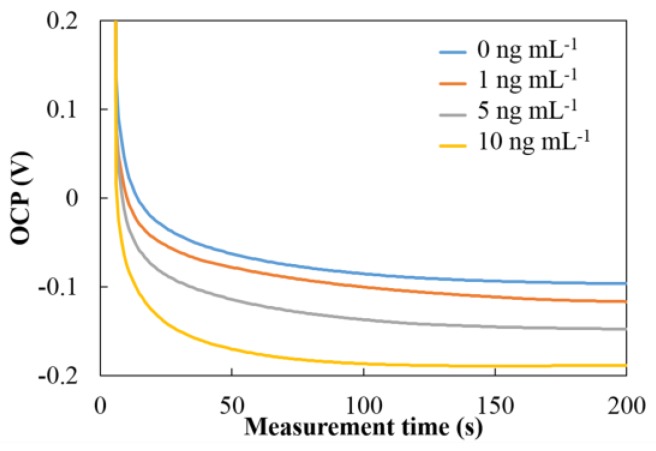
The open circuit potential (OCP) signal of PtNP-labeled immunocomplexes in 50 mM PBS pH 6.0 containing 1 mM hydrazine solution at different concentration of hCG.

The OCP was used to measure the hCG concentration after preparing the sandwich-type immunosensor. The number of PtNPs at secondary Mab was dependent on the hCG concentration and directly detected in 1 mM hydrazine solution. The different amounts of PtNPs on the electrode’s surface affected the catalytic activities towards the oxidation of hydrazine that result in the shift of potential of OCP as shown in Figure 4. The potential was shifted towards the negative direction with increasing hCG concentration. When hydrazine solution was added to the surface of the immunosensor containing PtNP-labeled secondary antibodies, the electrocatalysis of hydrazine oxidation occurred on the surfaces of the PtNPs. The overall oxidation current on the PtNPs, together with that on the working electrode, became larger than the overall reduction current. To maintain the OCP condition, the OCP shifts negatively to produce a zero net current. Figure 5 shows the OCP signal of PtNP-labeled immunocomplexes immobilized on SPCE at different concentrations of hCG. These results show that the oxidation of hydrazine was effectively electrocatalyzed on the surface of PtNPs and that the proposed system using the PtNP-based OCP immunosensors was successful for the determination of hCG. 

### 3.3. Optimization of Experimental Parameters

The pH of the medium solution of PBS and the concentration of hydrazine were studied because these factors can have an effect on the analytical detection results. These data were plotted as a function of the studied parameters. Next, the net OCP signal, after background subtraction, was measured, and the signal-to-blank difference (S-B) was plotted with respect to the studied parameters.

#### 3.3.1. Effect of pH of the Buffer Solution

**Figure 6:**
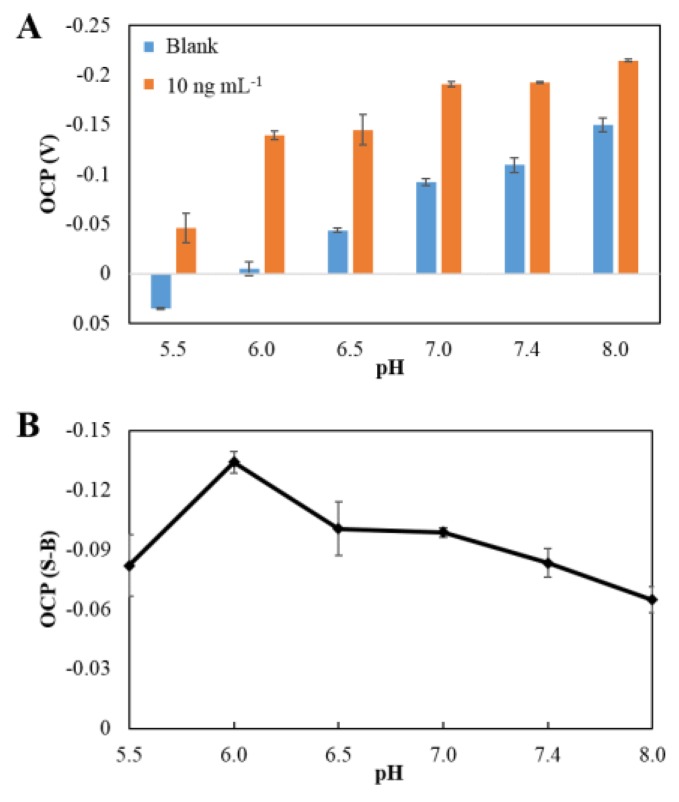
(**A**) The effect of pH of 50 mM PBS on the OCP signal of the blank (0 ng mL^−1^ of hCG: blue) and 10 ng mL^−1^ of hCG (orange); and (**B**) the plot of pH against OCP signal after background subtraction in 1 mM hydrazine solution.

The influence of the pH of the phosphate solution was investigated between pH 5.5 and 8.0. This range was selected because it is consistent with that found in the human body, as well as being appropriate for electrocatalytic oxidation of hydrazine at a concentration of 1 mM. The OCP signals were significantly shifted toward negative potential with the increase of pH in both the presence and absence of the PtNPs in the system shown in Figure 6A. The more positive potential at lower pH resulted from the protonated state of hydrazine (hydrazinium, N_2_H_5_^+^) under low pH (pKa of hydrazine is 7.9). The OCP shifted more rapidly to a negative potential in the presence rather than the absence of PtNPs due to the electrocatalytic oxidation of hydrazine at the surface of the PtNPs. The net OCP signal as a function of pH after background subtraction was plotted, as shown in Figure 6B; the highest differential OCP signal was observed at pH 6.0. Therefore, a phosphate solution of pH 6.0 was chosen as the optimal medium solution in further experiments.

#### 3.3.2. Concentration of Hydrazine Solution

**Figure 7:**
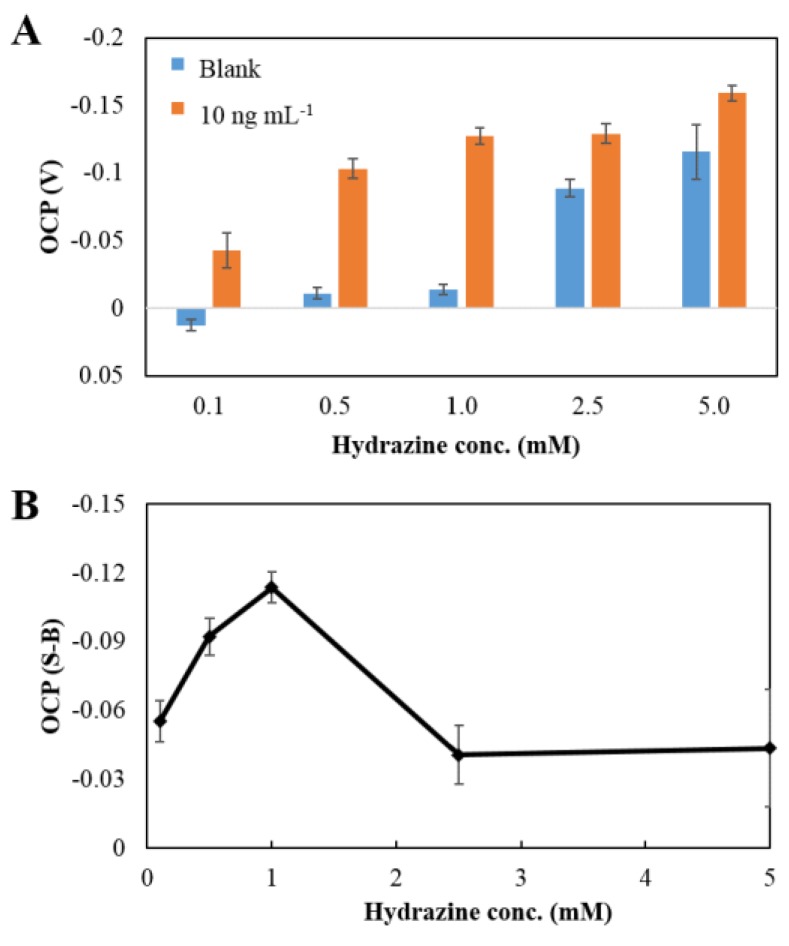
(**A**) The effect of the concentration of hydrazine in 50 mM PBS pH 6.0 on the OCP signal of a blank (0 ng mL^−1^ of hCG: blue) and 10 ng mL^−1^ of hCG (orange), and (**B**), the plot of the concentration of hydrazine against the OCP signal after background subtraction.

Not only the change in pH, but also the concentration of the hydrazine solution, may affect the OCP signal. The signal was shifted to a negative potential with an increasing concentration of hydrazine because of self-electrocatalytic oxidation of hydrazine at the surface of the electrode. The OCP signal shifted more significantly in the presence than in the absence of PtNPs (Figure 7A). The dependence of the OCP signal on the concentration of hydrazine after background subtraction was studied, as shown in Figure 7B. The highest OCP signal was observed at a concentration of 1 mM. Accordingly, the concentration of 1 mM was chosen as the optimal condition for further experiments.

The optimal conditions determined during the optimization are summarized in Table 1. These conditions were used for the detection by the OCP measurement of hCG using the PtNP-labeled immunocomplexes immobilized on SPCE.

### 3.4. Analytical Performance

**Figure 8:**
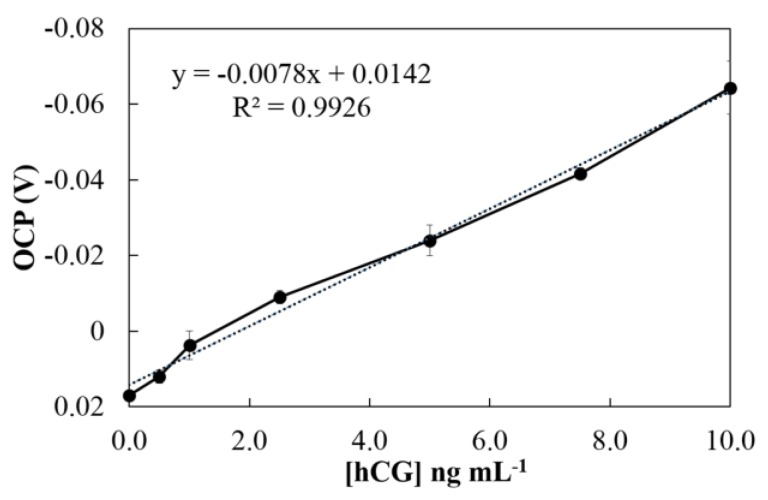
The calibration curve between the concentration of hCG and OCP signal under optimal conditions.

hCG was detected under optimal conditions using our proposed OCP system. A calibration curve was constructed by plotting the OCP signal versus known concentrations of hCG. Linearity was observed between 0 and 10 ng mL^−1^, with correlation coefficient (R^2^) higher than 0.99 (Figure 8). The LoD was calculated from 3 S_bl_/S, where S_bl_ is the standard deviation of blank measurement (*n* = 10), and S is the sensitivity of the method (slope of linearity). The LoD was found to be 0.28 ng mL^−1^ (28 mIU mL^−1^), when the performance of the proposed electrochemical immunoassay was compared to the previous electrochemical immunoassay using voltammetry and amperometry for the detection of hCG [9,10,11] as shown in Table 2. Although the previous techniques provided lower LoDs, the proposed system offers simpler electrochemical detection, a lower minimum amount of sample, and a more cost-effective disposable SPCE. To compare proposed sensor to the other hCG biosensors, such as lateral flow test strips colorimetric biosensor [20,21], our proposed sensor offers less amount of sample volume (as small as 2 μL), and ability to detect multi targets simultaneously with less interference. In addition, depending on the amount of hCG in pregnancy, marijuana use, hypogonadism (testicular failure), cirrhosis, inflammatory bowel disease, and duodenal ulcers, the obtained LoD is low enough to screen hCG concentration in clinical diagnostic applications [22]. The reproducibility of the proposed immunosensor was evaluated from the response to 10 ng mL^−1^ hCG at five independently prepared electrodes. The relative standard deviation (RSD) was 4.92%. Therefore, our proposed PtNP-based method demonstrates that it is a simpler electrochemical detection procedure with an acceptable sensitivity and reproducibility. Moreover, it could be applied to a simplified and miniaturized diagnostic system with minimal user manipulation.

This method is considered to be suitable for rapid multi-target measurement. When multi-working electrodes for different analytes were put into a sample solution, the OCP of each working electrode is established independently after hydrazine application. Therefore, after 5 min, all measurements data are obtained just by only one voltage measurements for each electrode. It is much easier compared to conventional DPV, where scanning of specific potential for each target is required. 

To verify the applicability of the proposed sensor, hCG in urine sample was analyzed. The standard addition method was used to investigate the practical applicability of the sensor. The concentrations of hCG were determined from the calibration curve. In the standard addition, the estimated values were in good agreement with the added a concentration of hCG, and a recovery experiment was used to evaluate the accuracy of the sensor (Table 3). The RSDs and recoveries were found in the ranges of 2.1–4.4% and 100.9–104.1%, respectively. Thus, the results clearly indicated the ability to measure hCG in real biological samples.

## 4. Conclusions

A new simple electrochemical immunoassay based on PtNPs was developed, using OCP as the detection method, to simplify the electrochemical detection of protein biomarkers. The electrocatalytic property of hydrazine on the surface of PtNPs simplifies the process of measuring potential. The change of signal was observed by direct electric detection by the OCP method without any external power source. The main advantages for the proposed immunosensor are in terms of the small sample volume, its low-cost, and its disposability. Moreover, our proposed PtNP-based detection system enabled a simple procedure for detecting biomarkers with acceptable sensitivity and reproducibility. 

## Figures and Tables

**Table 1:** Optimal detection parameters.

Parameters	Optimal
pH	6.0
Concentration of hydrazine	1 mM

**Table 2:** Comparison of the reported sensors and proposed sensor for the determination of hCG in urine sample.

Ref.	Method	Electrode	Linearity (ng mL^−1^)	LoD (ng mL^−1^)	Sample Volume (μL)
This work	OCP	SPCE	0.05–10	0.28	2
[9]	DPV	SPCE	0–2	0.036	2
[10]	Amperometry	Pt–Au alloy nanotube array	2.5–40	1.2	500
[11]	DPV	gold–silicon carbide nanocomposites	0.01–55–100	0.0042	≥500

**Table 3:** Determination of hCG in urine sample (*n* = 3).

Sample	Added (ng mL^−1^)	Detected (ng mL^−1^)	RSD (%)	Recovery (%)
Urine	1	1.04 ± 0.02	2.1	103.8
	3	3.12 ± 0.10	3.1	104.1
	5	5.02 ± 0.22	4.4	100.5
	7	7.08 ± 0.13	1.8	101.1
	9	9.08 ± 0.20	2.2	100.9

## References

[1] Bakker E., Qin Y. (2006). Electrochemical Sensors. Anal. Chem..

[2] Bannister J.V. (1991). Development of biosensors for immunoassays. Annali Dell’istituto Superiore di Sanita.

[3] Halsall H.B., Heineman W.R. (1990). Electrochemical immunoassay: An ultrasensitive method. J. Int. Fed. Clin. Chem..

[4] Wan Y., Su Y., Zhu X., Liu G., Fan C. (2013). Development of electrochemical immunosensors towards point of care diagnostics. Biosens. Bioelectron..

[5] Warsinke A., Benkert A., Scheller F.W. (2000). Electrochemical immunoassays. Fresenius’ J. Anal. Chem..

[6] Yan F., Wu J., Tan F., Yan Y., Ju H. (2009). A rapid and simple method for ultrasensitive electrochemical immunoassay of protein by an electric field-driven strategy. Anal. Chim. Acta.

[7] Kokkinos C., Economou A., Prodromidis M.I. (2016). Electrochemical immunosensors: Critical survey of different architectures and transduction strategies. TrAC Trends Anal. Chem..

[8] Ding L., Bond A.M., Zhai J., Zhang J. (2013). Utilization of nanoparticle labels for signal amplification in ultrasensitive electrochemical affinity biosensors: A review. Anal. Chim. Acta.

[9] Idegami K., Chikae M., Kerman K., Nagatani N., Yuhi T., Endo T., Tamiya E. (2008). Gold Nanoparticle-Based Redox Signal Enhancement for Sensitive Detection of Human Chorionic Gonadotropin Hormone. Electroanalysis.

[10] Tao M., Li X., Wu Z., Wang M., Hua M., Yang Y. (2011). The preparation of label-free electrochemical immunosensor based on the Pt–Au alloy nanotube array for detection of human chorionic gonadotrophin. Clin. Chim. Acta.

[11] Yang L., Zhao H., Fan S., Deng S., Lv Q., Lin J., Li C.-P. (2014). Label-free electrochemical immunosensor based on gold–silicon carbide nanocomposites for sensitive detection of human chorionic gonadotrophin. Biosens. Bioelectron..

[12] Videla H.A. (1996). Manual of Biocorrosion.

[13] Baker P.W., Ito K., Watanabe K. (2003). Marine prosthecate bacteria involved in the ennoblement of stainless steel. Environ. Microbiol..

[14] Dumańska J., Maksymiuk K. (2001). Studies on Spontaneous Charging/Discharging Processes of Polypyrrole in Aqueous Electrolyte Solutions. Electroanalysis.

[15] Song Y., Su D., Shen Y., Liu H., Wang L. (2017). Design and preparation of open circuit potential biosensor for in vitro and in vivo glucose monitoring. Anal. Bioanal. Chem..

[16] Ahammad A.J.S., Choi Y.-H., Koh K., Kim J.-H., Lee J.-J., Lee M. (2011). Electrochemical Detection of Cardiac Biomarker Troponin I at Gold Nanoparticle-Modified ITO Electrode by Using Open Circuit Potential. Int. J. Electrochem. Sci..

[17] Wong L.C.C., Jolly P., Estrela P. (2017). Development of a Sensitive Multiplexed Open Circuit Potential System for the Detection of Prostate Cancer Biomarkers. BioNanoScience.

[18] Zhou H., Park J.H., Fan F.-R.F., Bard A.J. (2012). Observation of Single Metal Nanoparticle Collisions by Open Circuit (Mixed) Potential Changes at an Ultramicroelectrode. J. Am. Chem. Soc..

[19] Xuan Viet N., Chikae M., Ukita Y., Maehashi K., Matsumoto K., Tamiya E., Hung Viet P., Takamura Y. (2013). Gold-linked electrochemical immunoassay on single-walled carbon nanotube for highly sensitive detection of human chorionic gonadotropinhormone. Biosens. Bioelectron..

[20] Nagatani N., Tanaka R., Yuhi T., Endo T., Kerman K., Takamura Y., Tamiya E. (2006). Gold nanoparticle-based novel enhancement method for the development of highly sensitive immunochromatographic test strips. Sci. Technol. Adv. Mater..

[21] Fu E., Liang T., Houghtaling J., Ramachandran S., Ramsey S.A., Lutz B., Yager P. (2011). Enhanced sensitivity of lateral flow tests using a two-dimensional paper network format. Anal. Chem..

[22] Berger P., Sturgeon C. (2014). Pregnancy testing with hCG—Future prospects. Trends Endocrinol. Metab..

